# Heat-Induced Dolomitization of Amorphous Calcium Magnesium Carbonate in a CO_2_-Filled Closed System

**DOI:** 10.1021/acsomega.2c03258

**Published:** 2022-11-28

**Authors:** Shingo Sugawara, Wataru Fujiya, Hiroyuki Kagi, Akira Yamaguchi, Ko Hashizume

**Affiliations:** † Faculty of Science, Ibaraki University, 2-1-1 Bunkyo, Mito, Ibaraki 310-8512, Japan; ‡ Geochemical Research Center, Graduate School of Science, The University of Tokyo, 7-3-1 Hongo, Tokyo 113-0033, Japan; § National Institute of Polar Research, 10-3 Midori-cho, Tachikawa, Tokyo 190-8518, Japan

## Abstract

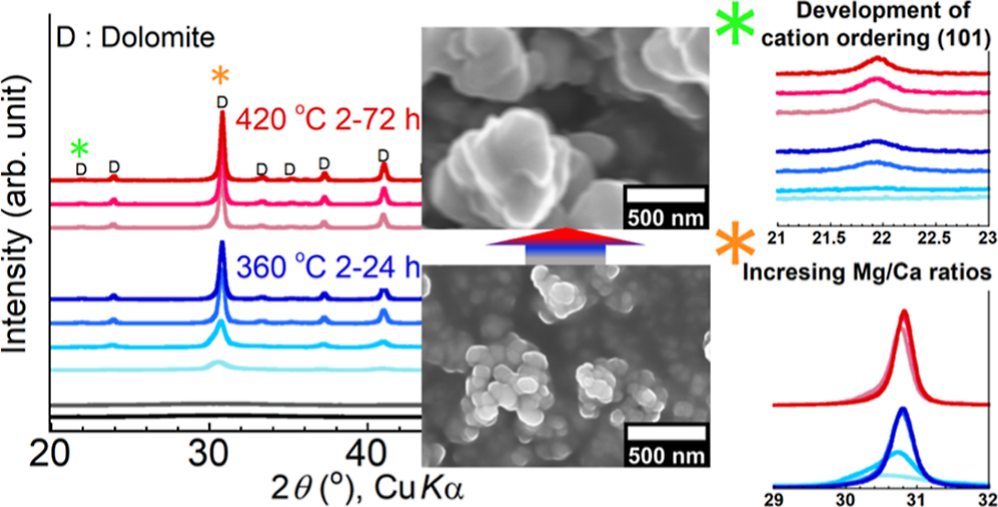

We report a method to synthesize dolomite [CaMg(CO_3_)_2_] from amorphous calcium magnesium carbonate (ACMC) via solid-state transformation. When ACMC is heated in air, it does not crystallize into dolomite but decomposes into Mg calcite, magnesium oxide, and CO_2_. Hence, we heated ACMC in a closed system filled with CO_2_ gas (pCO_2_ >1.2 bar at 420 °C) and produced submicron-sized dolomite. Single-phase dolomite was obtained after dissolving impurities in the run products, such as northupite [Na_3_Mg(CO_3_)_2_Cl] and eitelite [Na_2_Mg(CO_3_)_2_], in water. Also, we investigated the crystallization process of dolomite by changing the heating temperature and heating time. Despite crystallization by solid-state transformation, the heated samples crystallized to dolomite via Ca-rich protodolomite with no ordering reflection of X-ray diffraction as previously observed for hydrothermal synthesis. The results demonstrated that this crystallization pathway is kinetically favored even in solid-state transformation and that the Ca-rich protodolomite phase preferentially crystallizes during heating, leading to phase separation from the amorphous phase. Therefore, the crystallization process via protodolomite as a precursor is a common mechanism in dolomite crystallization, suggesting the presence of kinetic barriers other than hydration of cations.

## Introduction

Dolomite [CaMg(CO_3_)_2_] is a rhombohedral carbonate commonly found in sedimentary rocks. Its structure consists of an ordered arrangement of alternating layers of Ca^2+^ and Mg^2+^ cations (cation ordering) separated by layers of CO_3_^2–^.^1,2^ Although most natural dolomites are thought to form as a diagenetic replacement of limestones or a primary precipitation in solution,^1,3^ the detailed formation processes remain poorly understood and are much discussed as a dolomite problem.^1−7^ The dolomite problem stems from the inability to identify the formation process of natural dolomites for the following reasons: dolomite rarely forms in modern sedimentary environments and it has not been synthesized at ambient conditions (∼25 °C, 1 bar).^1,5,8,9^

The lack of dolomite in modern sediments is probably rooted in kinetic limitations, including difficulty in creating a Ca–Mg ordered arrangement.^2^ Pina et al.^9^ reported the degree of cation ordering of sedimentary dolomites with various geological ages from Proterozoic to Holocene. The cation ordering in these dolomites is more developed in older samples and poorly developed in younger samples in about the last 30 Myr (especially in Holocene samples). In hydrothermal experiments, it is known that well-ordered and stoichiometric dolomite forms through a dissolution-reprecipitation reaction via a metastable precursor, that is, protodolomite.^1,2,6,10−14^ Protodolomite is nonstoichiometric and disordered and is referred to by some authors as very high magnesium calcite.^1,2,10,12^ Because protodolomite can be synthesized at near ambient temperatures,^15−17^ dolomitization may proceed via such precursors even in natural environments.

There is still much debate about the limiting factors for dolomite formation. Strong hydration of Mg ions in solution has been considered to inhibit dolomite formation.^1,4^ Experimental and computational studies have supported this argument.^18−23^ On the other hand, the differences in Ca and Mg ionic radii may be an inherent barrier in forming dolomite structures, as inferred from the experiment of dolomite synthesis in dry organic solvents and experimental and theoretical studies on double carbonates like BaMg(CO_3_)_2_ and PbMg (CO_3_)_2_.^22,24−26^

Amorphous carbonates (MCO_3_·*n*H_2_O, M: divalent metal cation) are thermodynamically metastable relative to crystalline carbonates and provide a low-energy pathway for carbonate mineralization.^21,27,28^ Amorphous calcium carbonate (ACC), the most studied amorphous carbonate, crystallizes into calcite via a dissolution-reprecipitation (recrystallization) process in solution or high humidity.^29−36^ These crystallization mechanisms typically occur at ambient temperature. On the other hand, ACC easily crystallizes into calcite by heating in air.^37^ This crystallization process is considered to proceed by solid-state transformation.^30,33,34^ The crystallization by solid-state transformation has a variety of applications, including the study of biominerals,^38,39^ selective crystallization into desired polymorphs (calcite^30,33,34^ or aragonite^40,41^), the addition of large amounts of impurities to crystals,^42−44^ reference materials,^45^ and synthesis of nanoporous calcite with a high specific surface area.^46^

Amorphous calcium magnesium carbonate (ACMC) with dolomite composition may be a precursor for dolomite formation.^11,17,21^ However, previous studies reported the decomposition of ACMC into Mg calcite, MgO, and CO_2_ at high temperatures,^21,47^ likely due to the high dissociation pressure (pCO_2_) of dolomite. This thermodynamic reaction prevents the crystallization of ACMC by heating at ca. 400 °C in air, although a recent study reported the dolomite synthesis from a thin film of ACMC by heating in air.^41^ On the other hand, ACMC does not crystallize into dolomite at ambient temperature because of the kinetic barriers.^11,17^ Therefore, most previous studies have conducted the dolomitization of ACMC via dissolution-reprecipitation in hydrothermal solution.^11,13^

Here, we investigated the crystallization of dolomite via solid-state transformation by heating ACMC in a closed system filled with CO_2_ gas, aiming to develop a new method for dolomite synthesis without solution. Through the study of dolomite synthesis via solid-state transformation, we investigated the crystallization pathway to explore possible precursors of stoichiometric dolomite and to shed light on the kinetic barriers for dolomite formation. Although our experimental conditions are far different from those of natural environments, our study may reveal the general characteristics of dolomite formation as observed in hydrothermal experiments.

## Materials and Methods

We produced ACMC with a dolomite composition in a manner similar to that established by previous studies.^21,37,48^ Because ACMC preferentially incorporates Ca^2+^ compared to Mg^2+^, probably due to the strong free energy of hydration of the Mg^2+^ ion, an ACMC sample with dolomite composition was prepared using a 10 mL, 0.2 M (Mg + Ca) Cl_2_ solution (Mg/Ca = 2.36) and a 10 mL, 0.2 M Na_2_CO_3_ solution.^17,21,47^ For the solvent, ultrapure water (Millipore, resistivity = 18 MΩ·cm at 25 °C) was used. These solutions were mixed after ice-cooling for 10 min. After mixing, the precipitate was immediately filtered using a membrane filter (0.45 μm) under vacuum. Then, the precipitate was washed with acetone and dried in a vacuum desiccator (∼0.02 MPa). Acetone plays a role in dehydration and affects the water content of ACMC. After drying, ACMC was dehydrated and subsequently heated at temperatures up to 420 °C and for up to 72 h in a stainless-steel vessel filled with >1.2 bar CO_2_ gas (see results and discussion section). In all experiments, the CO_2_ gas was filled by downward displacement under ambient conditions, and thus, the exact CO_2_ partial pressure is unknown. We performed powder X-ray diffraction (XRD) analysis and thermogravimetry and differential thermal analysis (TG–DTA) for the run products, observed them using a field-emission scanning electron microscope (FE-SEM), and finally measured their chemical compositions using an electron probe microanalyzer (EPMA). We carried out data processing of the XRD analysis with the Software SmartLab Studio II by Rigaku. We used crystallographic open database (COD) files for phase identification and indexing of the samples. The details of the analytical technique are given in the Supporting Information.

## Results and Discussion

### Characterization of the Synthesized ACMC by Thermal Analysis

**Figure 1:**
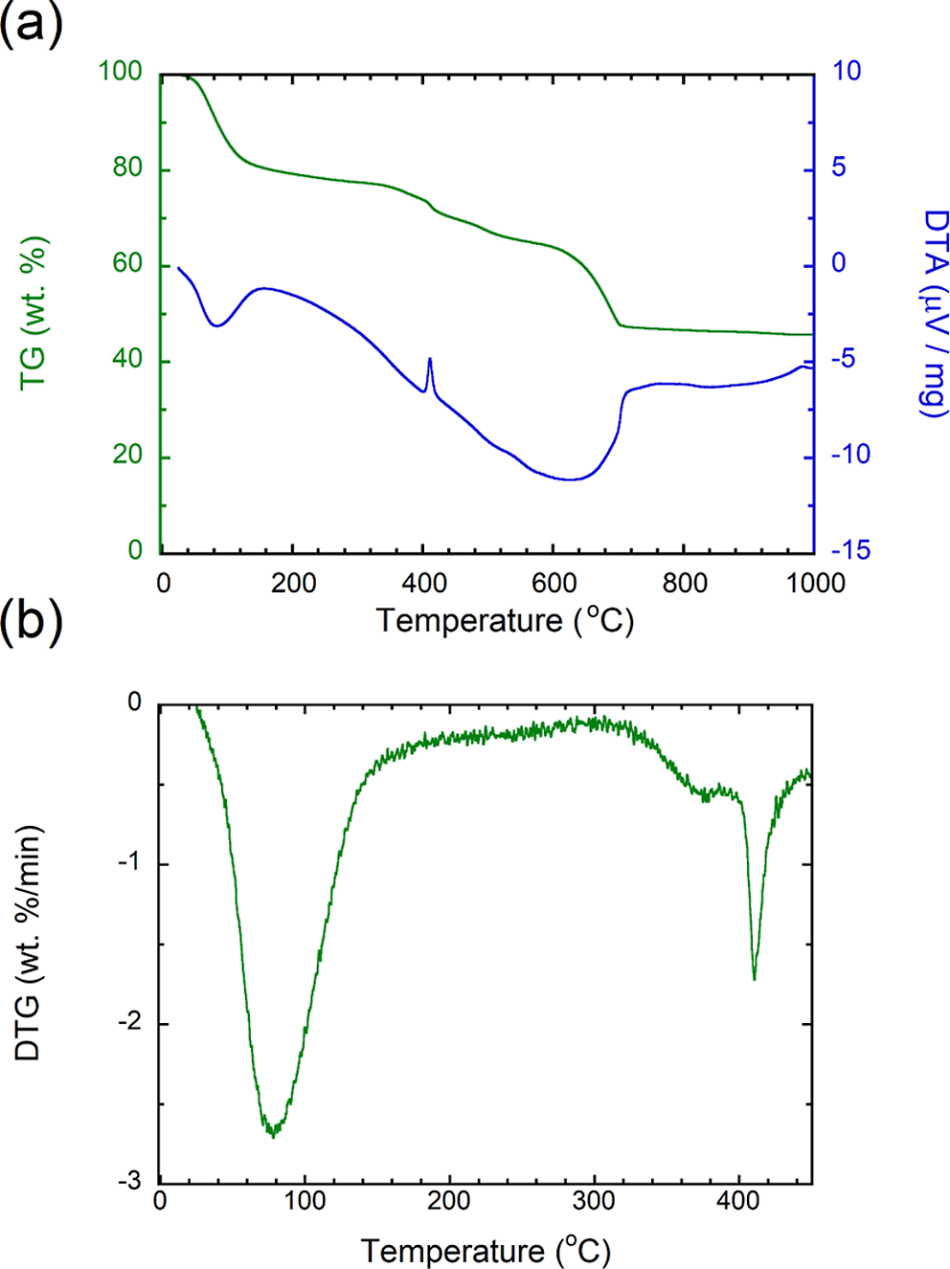
(a) TG and DTA curves of the synthesized ACMC. (b) DTG curve of the synthesized ACMC.

We used TG–DTA to characterize the ACMC and to examine the experimental condition for dolomitization of ACMC (Figure 1). The DTA curve showed an endothermic peak around 80 °C, a sharp exothermic peak at 410 °C, and a plateau above 700 °C (Figure 1a). These peaks indicate dehydration of ACMC, crystallization (of Mg calcite^21^), and the end of the decomposition reaction into oxide. The TG curve showed multiple mass loss steps, consistent with previous studies.^21,47^ The large mass loss that occurred around 80 and 700 °C was also detected in DTA as endothermic peaks, corresponding to the dehydration of ACMC and decomposition into CaO and CO_2_, respectively. The continuous mass loss from ∼100 to 400 °C probably suggests that both the dehydration of ACMC and decomposition of ACMC and/or Mg calcite occurred. The end of dehydration and the beginning of decomposition were not distinguished from the TG and DTA curves. Thus, the temperature at which the decomposition reaction began was estimated from differential TG (DTG) curve (Figure 1b). The DTG curve showed a large mass loss around 80 °C due to dehydration, and the mass loss rate approached zero until ∼310 °C. Then, the mass loss rate increased again above 310 °C and had a peak at 410 °C (the same temperature as the crystallization of Mg calcite).

The increase in the mass loss rate above 310 °C may indicate the beginning of the decomposition of ACMC and/or Mg calcite into MgO and CO_2_. TG and differential scanning calorimetry (TG–DSC) with Fourier transform infrared spectroscopy analyses of ACMC with dolomite composition (Ca_0.53_Mg_0.47_CO_3_) by Radha et al.^21^ demonstrated an endothermic peak due to the decomposition just before an exothermic peak due to the crystallization, along with a CO_2_ release around 400 °C, suggesting that the decomposition began before the crystallization. Therefore, the DTG data in this study suggest that the synthesized ACMC was almost completely dehydrated by 310 °C and the decomposition began above 310 °C.

The synthesized ACMC contained water, which must be removed before the heating experiments in a closed CO_2_ atmosphere. Based on the TG–DTA measurement, the synthesized ACMC was dehydrated under vacuum at 300 °C, the temperature at which it did not decompose. The synthesized ACMC crystallized to Mg calcite around 410 °C, as inferred from the sharp peak of the DTA curve (Figure 1a). The dissociation pressure calculated from Gibbs free energies of each phase given by Robie et al.^49^ is ∼1.2 bar at 427 °C, assuming that the decomposition reaction is CaMg(CO_3_)_2_ → CaCO_3_ + MgO + CO_2_. Hence, we heated the dehydrated ACMC at 420 °C for 72 h in a closed system filled with >1.2 bar CO_2_ gas (the upper limit of CO_2_ partial pressure at 420 °C calculated from the ideal gas law is 2.5 bar).

### Crystallization of Submicron-Sized Dolomite via ACMC

**Figure 2:**
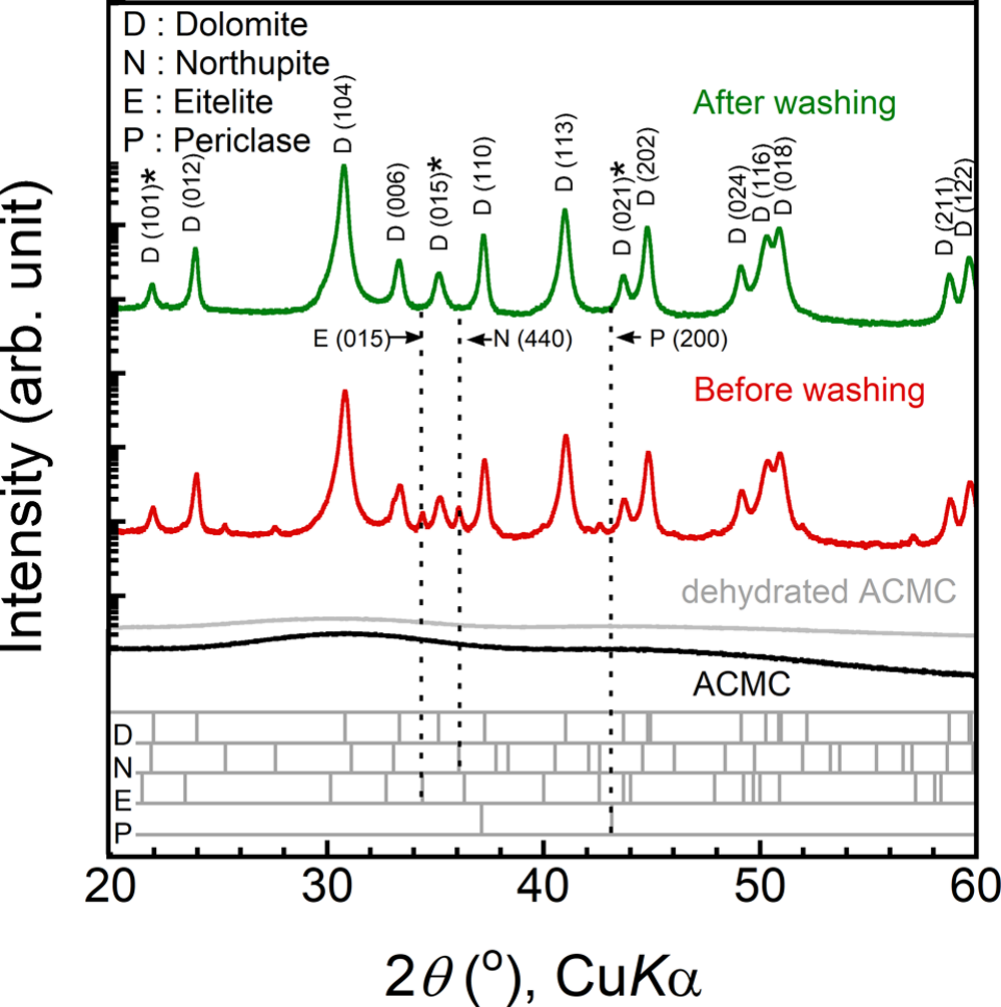
XRD patterns in the logarithmic scale of the ACMC (unheated), ACMC dehydrated at 300 °C, and the heated samples (420 °C, 72 h) before and after washing with ultrapure water. Before washing, the faint presence of northupite and eitelite can be found, but they disappeared after washing. We carried out phase identification and indexing of the XRD patterns using the COD file 9004933 (dolomite), 9009581 (northupite [Na_3_Mg(CO_3_)_2_Cl]), 9000298 (eitelite [Na_2_Mg(CO_3_)_2_]), and 9006786 (periclase [MgO]). The cation ordering reflections are indicated by asterisks.

We performed powder XRD analyses of the ACMC, dehydrated ACMC, and heated ACMC at 420 °C for 72 h (Figure 2). The XRD pattern of the ACMC did not exhibit any distinct diffraction peaks but broad halos confirming that it is an amorphous material. The XRD pattern of the dehydrated ACMC exhibits no clear diffraction peaks, indicating that it remained amorphous and neither crystallization nor decomposition occurred.

The XRD patterns of the heated sample at 420 °C for 72 h are consistent with those expected for dolomite (COD ID: 9004933).^50^ In addition, none of them contained magnesium oxide (MgO) (COD ID: 9006786).^51^ Therefore, the CO_2_ atmosphere suppressed the decomposition of ACMC at high temperatures. However, the heated samples typically contained other minerals such as northupite [Na_3_Mg(CO_3_)_2_Cl] (COD ID: 9009581) and eitelite [Na_2_Mg(CO_3_)_2_] (COD ID: 9000298) (Figure 2).^52,53^ The Na and Cl in these minerals were supplied from the reagents used for the synthesis. To dissolve these water-soluble impurities, we washed the heated sample in ultrapure water at ambient temperature for 10 min using an ultrasonic cleaner. The XRD measurement of the washed sample revealed that all impurities disappeared, and single-phase dolomite was successfully obtained (Figure 2). We measured the Mg/Ca ratio of the single-phase dolomite (0.83 ± 0.04, 2SD, corresponding to MgCO_3_ of 45.3 mol %) using an EPMA.

Note that the synthesized Ca-rich dolomite is thermodynamically unstable relative to stoichiometric dolomite.^54^ The Ca-rich dolomite as the final product in our study may have resulted from the initial ACMC composition. Therefore, stoichiometric dolomite could be synthesized by adjusting the Mg/Ca ratio of the starting solution. Moreover, amorphous phases incorporate various elements incompatible to carbonate due to a more flexible structure than crystalline phases. Matsunuma et al.^42^ showed that pressure-induced crystallization of ACC is more efficient for doping incompatible elements into crystals than crystallization in supersaturated solutions. Recently, calcite incorporating large amounts of incompatible elements such as Sr^2+^, Ba^2+^, and U^6+^ was synthesized by heat-induced crystallization of element-doped ACC.^44,45^ The dolomitization by a solid-phase reaction from ACMC may also facilitate the synthesis of element-doped dolomite.

**Figure 3:**
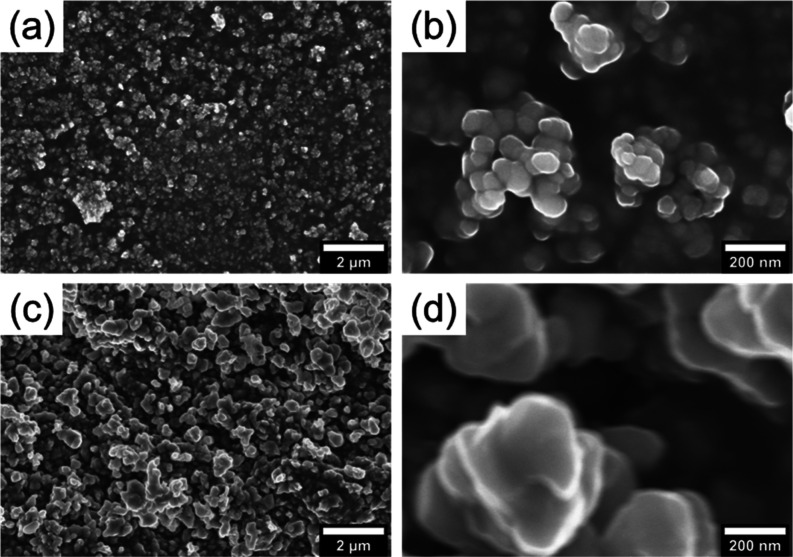
Secondary electron images of (a) dehydrated ACMC and (c) sample heated at 420 °C for 72 h in a closed system filled with CO_2_ gas. (b,d): Enlarged views of (a,c), respectively.

Figure 3 shows the secondary electron images of the dehydrated ACMC and the heated sample at 420 °C for 72 h taken with FE-SEM. The particles of the dehydrated ACMC were spherical and smaller than 100 nm in size. Their sizes and morphologies are similar to those observed by previous studies of ACC,^27,34,43,55^ ACMC,^11,43^ and dehydrated ACC.^27,55^ The particle size of the sample heated at 420 °C for 72 h was several 100 nm. Because there was essentially no residual water in the dehydrated ACMC, the morphological change by heating was likely due to agglomeration by sintering of small particles and the poorly developed crystal face resulting from solid-state transformation. Indeed, the morphology is very different from that of euhedral dolomite produced via dissolution-precipitation by hydrothermal synthesis,^11^ instead akin to that of the calcite crystallized from ACC via solid-state transformation (e.g., Figure S7 by Zou et al^34^).

### Dolomitization of ACMC via Protodolomite

**Figure 4:**
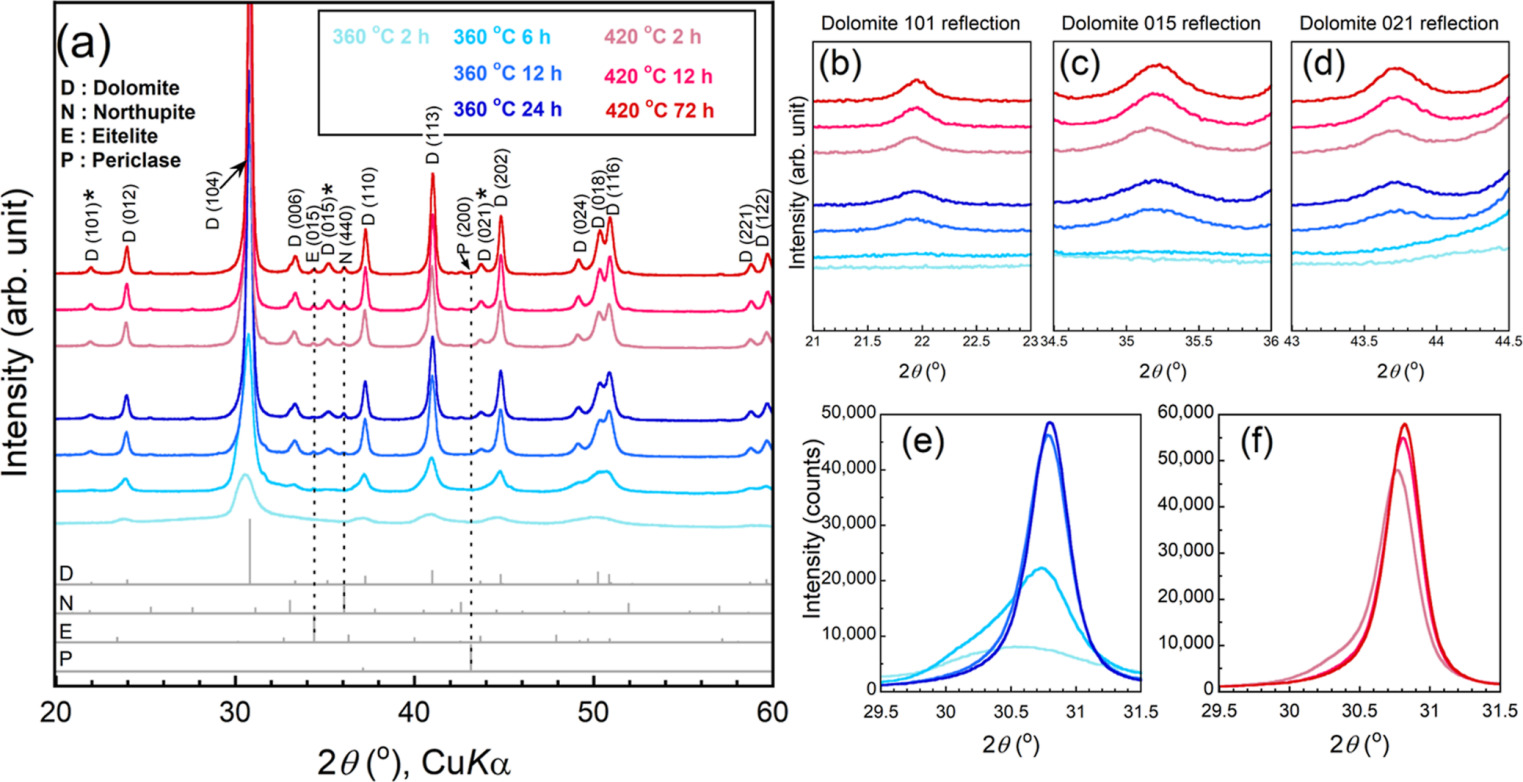
(a) XRD patterns of the samples heated in a closed system filled with CO_2_ gas. (b–d) Enlarged views of (a) focusing on ordering reflections (101, 015, and 021 reflections) of dolomite. (e–f) Enlarged views of (a) focusing on peak shifts, where absolute intensities are shown. The phase identification and indexing of the XRD patterns were carried out using COD files 9004933 (dolomite), 9009581 (northupite [Na_3_Mg(CO_3_)_2_Cl]), 9000298 (eitelite [Na_2_Mg(CO_3_)_2_]), and 9006786 (periclase [MgO]). The cation ordering reflections are indicated by asterisks.

To investigate the dolomitization process of ACMC via solid-state transformation, we heated the dehydrated ACMC at 360 °C for 2, 6, 12, and 24 h, and at 420 °C for 2, 12, and 72 h in a closed system filled with >1.2 bar CO_2_ gas (the upper limits of CO_2_ partial pressure at 360 and 420 °C calculated from the ideal gas law are 2.3 bar and 2.5 bar, respectively). The XRD pattern of the sample heated at 360 °C for 2 h is consistent with protodolomite,^2,11,13,17^ which does not show 101, 015, and 021 reflections, corresponding to the absence of cation ordering (Figures 4a–e, S1, and Table 1). On the other hand, the 101, 015, and 021 ordering reflections were observed for the samples heated at 360 °C for 12–24 h and 420 °C for 2–72 h, indicating that these samples crystallized to dolomite (Figure 4a–d). It should be stressed that the diffraction peaks of the heated samples were higher and sharper and shifted toward higher angles (Figure 4e,f and Table 1) with increasing heating temperature and/or heating time.

**Table 1:** Peak Positions of the 104 Reflection and the Degree of Cation Ordering in the XRD Patterns (Figure 4), and the MgCO_3_ Concentrations Estimated from the 104 Peak Positions (See Text)

temp (°C)	time (h)	2θ	*d* (104)	ordering ratio (015/110)	MgCO_3_ (mol %)
360	2	30.55	2.92	n.d.^a^	37
360	6	30.64	2.92	n.d.^a^	40
360	12	30.75	2.91	0.14	44
360	24	30.76	2.90	0.15	44
420	2	30.73	2.91	0.11	43
420	12	30.77	2.90	0.18	44
420	72	30.78	2.90	0.19	45
					
COD ID: 9004933		30.81	2.90	0.41	45

a n.d. = not detectable.

The peak shifts may indicate that the lattice volume decreased with increasing Mg/Ca ratios of dolomite. This is supported by the fact that these shifts were not observed for calcite transformed from ACC (Figure S2). The peak positions did not change significantly among the samples heated at 420 °C for 12–72 h. This suggests that all amorphous phases crystallized, and dolomitization was completed by 12 h.

Because the peak position of 104 reflection of dolomite depends on the Mg/Ca ratios,^2,56^ the mole fraction of MgCO_3_ of dolomite can be estimated semiquantitatively by the following equation: MgCO_3_ mol % = 100 – (333.33*d*_104_ – 911.99), where *d*_104_ represents the *d*-spacing (Å) of the 104 reflection.^57^ Based on the above equation, the concentration of MgCO_3_ in the samples is inferred to have increased from 37 to 45 mol % with increasing heating temperature and heating time (Table 1). The concentration of MgCO_3_ in the sample heated at 420 °C for 72 h is consistent with the value measured by the EPMA (45.3 mol %).

The dolomitization by hydrothermal synthesis, where a dissolution-reprecipitation reaction takes place, is known to occur via protodolomite lacking in 101, 015, and 021 reflection peaks unique to dolomite.^11,12,58^ Protodolomite typically has nonstoichiometric Mg/Ca ratios smaller than unity.^11,12,58^ We observed similar features for the dolomitization in this study, such as the increase of the Mg/Ca ratios and the development of 101, 015, and 021 reflection peaks upon increasing the heating temperature and/or heating time. Therefore, it is likely that the dolomitization of ACMC by heating without solution is also via protodolomite.

### Cation Ordering

The degree of cation ordering in dolomite can be estimated semiquantitatively from the intensity ratio of 015 ordering reflection to nearby 110 reflection irrelevant to cation ordering.^2,59^ The 015 reflection intensity of a fully ordered dolomite should be comparable to the 110 reflection intensity.^2^ In this study, the intensity ratio of the 015–110 reflections increased with the heating time, although it was only ∼0.2 at 420 °C for 72 h (Table 1).

The low degree of ordering may be due to the nonstoichiometric composition of the synthesized dolomite and/or the reaction time insufficient for the ordered arrangement of alternating layers of Ca^2+^ and Mg^2+^. The natural Ca–Mg carbonates formed in Holocene display 015/110 intensity ratios of <0.4 or lack cation ordering, even if these samples have near-dolomite stoichiometry.^9^ On the other hand, dolomites in older sediments have 015/110 intensity ratios from 0.4 to 1.0, even if their compositions are far different from dolomite stoichiometry.^9^ Therefore, the low ordering degree of our samples is most likely due to the short reaction time.

On the other hand, hydrothermal synthesis develops cation ordering in a shorter time. For example, Kaczmarek and Sibley performed the hydrothermal synthesis of dolomite by heating calcite in Mg–Ca–Cl-solution with a Mg/Ca ratio of 1.27 at 218 °C.^58^ Although they took roughly the same time as that for the solid-phase reaction in our study, the 015/110 intensity ratios of dolomite produced by them were ∼0.5. Moreover, as the reaction time increased to 120 h, the intensity ratio of 015–110 reflection increased to 0.7. In our study, the ordering developed slowly in spite of the higher reaction temperature than that for hydrothermal synthesis. These observations suggest that the ordering develops slowly during the crystallization by the solid-state transformation compared with the dissolution-reprecipitation process of hydrothermal synthesis.

### Implications for the Crystallization of Dolomite in Natural Environments

In current sedimentary environments, natural dolomite rarely occurs.^1,3^ Moreover, the synthesis of dolomite from a solution has not been achieved at ambient temperatures.^2,8,11,17^ Nonstoichiometric and disordered protodolomite is known to form as a reaction intermediate in hydrothermal environments.^1,2,10−13^ It should be noted that well-ordered dolomite is more common in older sedimentary rocks; on the other hand, poorly ordered dolomite is predominantly found in younger ones.^9^ Protodolomite was also observed in laboratory experiments where dolomite was produced in solution at near ambient temperatures.^15−17^

The present work revealed that ACMC likely crystallizes into dolomite via protodolomite even in solid-state transformation by heating in CO_2_ atmosphere. Our results and the observations mentioned above might suggest that dolomite formation via protodolomite, which seems kinetically favored, commonly occurs irrespective of the formation pathways (i.e., dissolution-reprecipitation, solid-state transformation, or primary precipitation) and starting materials (i.e., crystalline or amorphous carbonates), and dolomite cannot form directly. This argument is consistent with the conclusions from the laboratory experiment on the synthesis of a dolomite analogue, norsethite [BaMg(CO_3_)_2_], which demonstrated that fully ordered norsethite formed through a number of precursors.^24^

Because ACMC did not directly crystallize to dolomite in a solid-state transformation, we infer that the hydration of Mg ions is not the sole reason for the kinetic inhibition of dolomite formation and that the dolomite structures with different ionic radii of Mg and Ca could be an inherent barrier in forming dolomite. Xu et al.^22^ showed that protodolomite rather than dolomite formed in a dry organic solvent, that is, in the absence of water. This suggests the presence of a fundamental barrier other than cation hydration. Furthermore, a theoretical simulation and experiment of the synthesis of double carbonates suggested that double carbonates with smaller radii ratios of cations to Mg (X/Mg: X = Ca, Sr, Pb, Ba, or Ra) are more difficult to synthesize.^26^

### Implications for the Nature of ACMC

As mentioned above, our study suggests that the Mg/Ca ratios of the heated samples increased when increasing the heating temperature and/or the heating time, as observed for the samples produced by hydrothermal synthesis. The dolomitization by heating in our study does not have any external Mg^2+^ source to increase the Mg/Ca ratios of the heated samples, whereas Mg^2+^ can be supplied from solution in the case of hydrothermal synthesis. Therefore, at the early stage of heating, the Ca in ACMC prefers to crystallize over Mg to produce protodolomite richer in Ca than the initial, and then it reacted with the remaining amorphous magnesium carbonate (AMC) or ACMC richer in Mg than the initial, progressively increasing its Mg/Ca ratio. Some XRD patterns of the heated samples indicated the possible presence of northupite and eitelite, which bear Mg (Figure S1). However, they likely did not contribute to the increase of the Mg/Ca ratio because the abundances of northupite and eitelite did not change significantly during the heating experiments.

Radha et al.^21^ performed the TG–DSC measurements of ACMC (MgCO_3_ >47 mol %) and found that the ACMC lost its mass in multiple steps during heating. They attributed the mass loss in multiple steps to the presence of two components in their samples, namely, ACMC and AMC, with different kinetics of dehydration reactions. They concluded that either (1) ACMC (MgCO_3_ <47 mol %) and AMC were present at the time of synthesis or (2) these phases separated from one phase during heating. We cannot rule out the possibility that two components were present at the synthesis time. However, it seems more likely that protodolomite crystallization process resulted in the separation of Mg-rich amorphous phase because the crystallization process by heating is similar to that by hydrothermal synthesis, and the dolomitization via protodolomite is known to be kinetically favored.

## Conclusions

We investigated the crystallization of dolomite via solid-state transformation by heating ACMC in a closed system filled with CO_2_ gas. The XRD patterns showed that the heated samples crystallized to dolomite, indicating that the CO_2_ atmosphere suppressed the decomposition of ACMC at high temperatures. The heated samples typically contain northupite and eitelite, although we can easily remove these impurities by washing them in ultrapure water to obtain single-phase dolomite. The XRD intensity ratio of the 015–110 reflections, which indicates the degree of cation ordering, was only ∼0.2 at 420 °C for 72 h, likely due to slow development by solid-state transformation. Then, the crystallization process of dolomite was investigated by changing the heating temperature and heating time. With increasing heating temperature and heating time, the dolomitization of ACMC with dolomite composition proceeded via a Ca-rich protodolomite phase with no ordering reflection. This suggests that the dolomitization via protodolomite is kinetically favored even under a dry condition, as previously observed in hydrothermal synthesis. Therefore, the crystallization of protodolomite as a precursor is a common process, suggesting that kinetic barriers other than hydration play a role in inhibiting dolomite formation. Also, these results indicate that the phase separation into Ca-rich protodolomite and a Mg-rich amorphous phase during heating of ACMC likely occurs.

## Supporting Information Available

XRD pattern of a run product including COD files used for phase identification and indexing of the samples, enlarged views of Figure 4, XRD patterns of the ACC and the samples heated in air, additional secondary electron images, and experimental details (PDF)

## References

[ref1] Warren J. Dolomite: Occurrence, Evolution and Economically Important Associations. Earth Sci. Rev. 2000, 52, 1–81. 10.1016/s0012-8252(00)00022-2.

[ref2] Gregg J. M.; Bish D. L.; Kaczmarek S. E.; Machel H. G. Mineralogy, Nucleation and Growth of Dolomite in the Laboratory and Sedimentary Environment: A Review. Sedimentology 2015, 62, 1749–1769. 10.1111/sed.12202.

[ref3] Machel H. G. Concepts and Models of Dolomitization – A Critical Reappraisal. In The Geometry and Petrogenesis of Dolomite Hydrocarbon Reservoirs; Braithwaite C. J. R., Rizzi G., Darke G., Eds.; Geological Society London Special Publications, 2004; Vol. 235, pp 7–63.

[ref4] Lippmann F. Sedimentary Carbonate Minerals; Minerals, Rocks and Mountains; Springer-Verlag: Berlin, Germany, 1973.

[ref5] Holland H. D.; Zimmermann H. The Dolomite Problem Revisited. Int. Geol. Rev. 2000, 42, 481–490. 10.1080/00206810009465093.

[ref6] Arvidson R. S.; Mackenzie F. T. The Dolomite Problem: Control of Precipitation Kinetics by Temperature and Saturation State. Am. J. Sci. 1999, 299, 257–288. 10.2475/ajs.299.4.257.

[ref7] Pina C. M.; Pimentel C.; Crespo A. The Dolomite Problem: A Matter of Time. ACS Earth Space Chem. 2022, 6, 1468–1471. 10.1021/acsearthspacechem.2c00078.

[ref8] Land L. S. Failure to Precipitate Dolomite at 25°C from Dilute Solution despite 1000-Fold Oversaturation after 32 Years. Aquat. Geochem. 1998, 4, 361–368. 10.1023/a:1009688315854.

[ref9] Pina C. M.; Pimentel C.; Crespo Á. Dolomite Cation Order in the Geological Record. Chem. Geol. 2020, 547, 119667 10.1016/j.chemgeo.2020.119667.

[ref10] Kell-Duivestein I. J.; Baldermann A.; Mavromatis V.; Dietzel M. Controls of Temperature, Alkalinity and Calcium Carbonate Reactant on the Evolution of Dolomite and Magnesite Stoichiometry and Dolomite Cation Ordering Degree–An Experimental Approach. Chem. Geol. 2019, 529, 119292 10.1016/j.chemgeo.2019.119292.

[ref11] Rodriguez-Blanco J. D.; Shaw S.; Benning L. G. A Route for the Direct Crystallization of Dolomite. Am. Mineral. 2015, 100, 1172–1181. 10.2138/am-2015-4963.

[ref12] Kaczmarek S. E.; Thornton B. P. The Effect of Temperature on Stoichiometry, Cation Ordering, and Reaction Rate in High-Temperature Dolomitization Experiments. Chem. Geol. 2017, 468, 32–41. 10.1016/j.chemgeo.2017.08.004.

[ref13] Müller I. A.; Rodriguez-Blanco J. D.; Storck J. C.; do Nascimento G. S.; Bontognali T. R. R.; Vasconcelos C.; Benning L. G.; Bernasconi S. M. Calibration of the Oxygen and Clumped Isotope Thermometers for (Proto-)Dolomite Based on Synthetic and Natural Carbonates. Chem. Geol. 2019, 525, 1–17. 10.1016/j.chemgeo.2019.07.014.

[ref14] Malone M. J.; Baker P. A.; Burns S. J. Recrystallization of Dolomite: An Experimental Study from 50–200 °C. Geochim. Cosmochim. Acta 1996, 60, 2189–2207. 10.1016/0016-7037(96)00062-2.

[ref15] Oomori T.; Kitano Y. Synthesis of Protodolomite from Sea Water Containing dioxin. Geochem. J. 1987, 21, 59–65. 10.2343/geochemj.21.59.

[ref16] Zhang F.; Xu H.; Konishi H.; Roden E. E. A Relationship between d104 Value and Composition in the Calcite-Disordered Dolomite Solid-Solution Series. Am. Mineral. 2010, 95, 1650–1656. 10.2138/am.2010.3414.

[ref17] Montes-Hernandez G.; Renard F.; Auzende A. L.; Findling N. Amorphous Calcium-Magnesium Carbonate (ACMC) Accelerates Dolomitization at Room Temperature under Abiotic Conditions. Cryst. Growth Des. 2020, 20, 1434–1441. 10.1021/acs.cgd.9b01005.

[ref18] Jiao D.; King C.; Grossfield A.; Darden T. A.; Ren P. Simulation of Ca^2+^ and Mg^2+^ Solvation Using Polarizable Atomic Multipole Potential. J. Phys. Chem. B 2006, 110, 18553–18559. 10.1021/jp062230r. 16970483

[ref19] Tommaso D.; de Leeuw N. H. Structure and Dynamics of the Hydrated Magnesium Ion and of the Solvated Magnesium Carbonates: Insights from First Principles Simulations. Phys. Chem. Chem. Phys. 2010, 12, 894–901. 10.1039/b915329b. 20066374

[ref20] Yang Y.; Sahai N.; Romanek C. S.; Chakraborty S. A Computational Study of Mg^2+^ Dehydration in Aqueous Solution in the Presence of HS– and Other Monovalent Anions – Insights to Dolomite Formation Geochim. Cosmochim. Acta 2012, 88, 77–87. 10.1016/j.gca.2012.03.018.

[ref21] Radha A. V.; Fernandez-Martinez A.; Hu Y.; Jun Y. S.; Waychunas G. A.; Navrotsky A. Energetic and Structural Studies of Amorphous Ca_1-X_Mg_X_CO_3_·nH_2_O (0≤x≤1). Geochim. Cosmochim. Acta 2012, 90, 83–95. 10.1016/j.gca.2012.04.056.

[ref22] Xu J.; Yan C.; Zhang F.; Konishi H.; Xu H.; Teng H. H. Testing the Cation-Hydration Effect on the Crystallization of Ca-Mg-CO_3_ Systems. Proc. Natl. Acad. Sci. U.S.A. 2013, 110, 17750–17755. 10.1073/pnas.1307612110. 24127571 PMC3816432

[ref23] Fang Y.; Zhang F.; Farfan G. A.; Xu H. Low-Temperature Synthesis of Disordered Dolomite and High-Magnesium Calcite in Ethanol–Water Solutions: The Solvation Effect and Implications. ACS Omega 2021, 7, 281–292. 10.1021/acsomega.1c04624. 35036699 PMC8757334

[ref24] Pimentel C.; Pina C. M. The Formation of the Dolomite-Analogue Norsethite: Reaction Pathway and Cation Ordering. Geochim. Cosmochim. Acta 2014, 142, 217–223. 10.1016/j.gca.2014.07.021.

[ref25] Pimentel C.; Pina C. M. Reaction Pathways towards the Formation of Dolomite-Analogues at Ambient Conditions. Geochim. Cosmochim. Acta 2016, 178, 259–267. 10.1016/j.gca.2015.12.040.

[ref26] Pimentel C.; Pina C. M.; Sainz-Díaz C. I. New Insights into Dolomite and Dolomite-Analogue Structures from First Principles Calculations. ACS Earth Space Chem. 2022, 6, 2360 10.1021/acsearthspacechem.2c00100. PMC9589907 36303719

[ref27] Radha A. V.; Forbes T. Z.; Killian C. E.; Gilbert P. U. P. A.; Navrotsky A. Transformation and Crystallization Energetics of Synthetic and Biogenic Amorphous Calcium Carbonate. Proc. Natl. Acad. Sci. U.S.A. 2010, 107, 16438–16443. 10.1073/pnas.1009959107. 20810918 PMC2944757

[ref28] Sel O.; Radha A. V.; Dideriksen K.; Navrotsky A. Amorphous Iron (II) Carbonate: Crystallization Energetics and Comparison to Other Carbonate Minerals Related to CO_2_ Sequestration. Geochim. Cosmochim. Acta 2012, 87, 61–68. 10.1016/j.gca.2012.03.011.

[ref29] Ogino T.; Suzuki T.; Sawada K. The Formation and Transformation Mechanism of Calcium Carbonate in Water. Geochim. Cosmochim. Acta 1987, 51, 2757–2767. 10.1016/0016-7037(87)90155-4.

[ref30] Xu X.; Han J. T.; Kim D. H.; Cho K. Two Modes of Transformation of Amorphous Calcium Carbonate Films in Air. J. Phys. Chem. B 2006, 110, 2764–2770. 10.1021/jp055712w. 16471883

[ref31] Rodriguez-Blanco J. D.; Shaw S.; Benning L. G. The Kinetics and Mechanisms of Amorphous Calcium Carbonate (ACC) Crystallization to Calcite, via Vaterite. Nanoscale 2011, 3, 265–271. 10.1039/c0nr00589d. 21069231

[ref32] Rodriguez-Blanco J. D.; Shaw S.; Bots P.; Roncal-Herrero T.; Benning L. G. The Role of pH and Mg on the Stability and Crystallization of Amorphous Calcium Carbonate. J. Alloys Compd. 2012, 536, 477–479. 10.1016/j.jallcom.2011.11.057.

[ref33] Ihli J.; Wong W. C.; Noel E. H.; Kim Y. Y.; Kulak A. N.; Christenson H. K.; Duer M. J.; Meldrum F. C. Dehydration and Crystallization of Amorphous Calcium Carbonate in Solution and in Air. Nat. Commun. 2014, 5, 3169 10.1038/ncomms4169. 24469266 PMC4085778

[ref34] Zou Z.; Bertinetti L.; Politi Y.; Jensen A. C. S.; Weiner S.; Addadi L.; Fratzl P.; Habraken W. J. E. M. Opposite Particle Size Effect on Amorphous Calcium Carbonate Crystallization in Water and during Heating in Air. Chem. Mater. 2015, 27, 4237–4246. 10.1021/acs.chemmater.5b00145.

[ref35] Konrad F.; Gallien F.; Gerard D. E.; Dietzel M. Transformation of Amorphous Calcium Carbonate in Air. Cryst. Growth Des. 2016, 16, 6310–6317. 10.1021/acs.cgd.6b00906.

[ref36] Du H.; Amstad E. Water: How Does It Influence the CaCO_3_ Formation?. Angew. Chem., Int. Ed. 2019, 59, 1798–1816. 10.1002/anie.201903662. 31081984

[ref37] Koga N.; Nakagoe Y.; Tanaka H. Crystallization of Amorphous Calcium Carbonate. Thermochim. Acta 1998, 318, 239–244. 10.1016/s0040-6031(98)00348-7.

[ref38] Politi Y.; Metzler R. A.; Abrecht M.; Gilbert B.; Wilt F. H.; Sagi I.; Addadi L.; Weiner S.; Gilbert P. Transformation Mechanism of Amorphous Calcium Carbonate into Calcite in the Sea Urchin Larval Spicule. Proc. Natl. Acad. Sci. U.S.A. 2008, 105, 17362–17366. 10.1073/pnas.0806604105. 18987314 PMC2582271

[ref39] Gong Y. U. T.; Killian C. E.; Olson I. C.; Appathurai N. P.; Amasino A. L.; Martin M. C.; Holt L. J.; Wilt F. H.; Gilbert P. U. P. A. Phase Transitions in Biogenic Amorphous Calcium Carbonate. Proc. Natl. Acad. Sci. U.S.A. 2012, 109, 6088–6093. 10.1073/pnas.1118085109. 22492931 PMC3341025

[ref40] Walker J. M.; Marzec B.; Nudelman F. Solid-State Transformation of Amorphous Calcium Carbonate to Aragonite Captured by Cryotem. Angew. Chem., Int. Ed. 2017, 56, 11740–11743. 10.1002/anie.201703158. PMC5656811 28742941

[ref41] Zhang S.; Nahi O.; Chen L.; Aslam Z.; Kapur N.; Kim Y. Y.; Meldrum F. C. Magnesium Ions Direct the Solid-State Transformation of Amorphous Calcium Carbonate Thin Films to Aragonite, Magnesium-Calcite, or Dolomite. Adv. Funct. Mater. 2022, 32, 2201394 10.1002/adfm.202201394.

[ref42] Matsunuma S.; Kagi H.; Komatsu K.; Maruyama K.; Yoshino T. Doping Incompatible Elements into Calcite through Amorphous Calcium Carbonate. Cryst. Growth Des. 2014, 14, 5344–5348. 10.1021/cg500953h.

[ref43] Albéric M.; Bertinetti L.; Zou Z.; Fratzl P.; Habraken W.; Politi Y. The Crystallization of Amorphous Calcium Carbonate Is Kinetically Governed by Ion Impurities and Water. Adv. Sci. 2018, 5, 1701000 10.1002/advs.201701000. PMC5980180 29876222

[ref44] Saito A.; Kagi H.; Marugata S.; Komatsu K.; Enomoto D.; Maruyama K.; Kawano J. Incorporation of Incompatible Strontium and Barium. Minerals 2020, 10, 270 10.3390/min10030270.

[ref45] Miyajima Y.; Saito A.; Kagi H.; Yokoyama T.; Takahashi Y.; Hirata T. Incorporation of U, Pb and Rare Earth Elements in Calcite through Crystallisation from Amorphous Calcium Carbonate: Simple Preparation of Reference Materials for Microanalysis. Geostand. Geoanal. Res. 2020, 45, 189–205. 10.1111/ggr.12367.

[ref46] Wang Q.; Zou Z.; Wang H.; Wang W.; Fu Z. Pressure-Induced Crystallization and Densification of Amorphized Calcium Carbonate Hexahydrate Controlled by Interfacial Water. J. Colloid Interface Sci. 2022, 611, 346–355. 10.1016/j.jcis.2021.12.095. 34959008

[ref47] Purgstaller B.; Goetschl K. E.; Mavromatis V.; Dietzel M. Solubility Investigations in the Amorphous Calcium Magnesium Carbonate System. CrystEngComm 2019, 21, 155–164. 10.1039/c8ce01596a. 30760969 PMC6336086

[ref48] Yoshino T.; Maruyama K.; Kagi H.; Nara M.; Kim J. C. Pressure-Induced Crystallization from Amorphous Calcium Carbonate. Cryst. Growth Des. 2012, 12, 3357–3361. 10.1021/cg2017159.

[ref49] Robie R. A.; Hemingway B. S.; Fisher J. R. Thermodynamic Properties of Minerals and Related Substances at 298.15 K and 1 Bar (10^5^ Pascals) Pressure and at Higher Temperatures (Reprinted with Corrections); US Geological Survey Bulletin 1452, 1979.

[ref50] Drits V. A.; McCarty D. K.; Sakharov B.; Milliken K. L. New insight into structural and compositional variability in some ancient excess-Ca dolomite. Can. Mineral. 2005, 43, 1255–1290. 10.2113/gscanmin.43.4.1255.

[ref51] Zhang J. Effect of Pressure on the Thermal Expansion of MgO up to 8.2 GPa. Phys. Chem. Miner. 2000, 27, 145–148. 10.1007/s002690050001.

[ref52] Negro A.; Giuseppetti G.; Tadini C. Refinement of the Crystal Structure of Northupite: Na_3_Mg(CO_3_)_2_Cl. Tschermaks Mineral. Petrogr. Mittl. 1975, 22, 158–163. 10.1007/bf01089114.

[ref53] Pabst A. The Crystallography and Structure of Eitelite, Na_2_Mg(CO_3_)_2_. Am. Mineral. 1973, 58, 211–217.

[ref54] Chai L.; Navrotsky A.; Reeder R. J. Energetics of Calcium-Rich Dolomite. Geochim. Cosmochim. Acta 1995, 59, 939–944. 10.1016/0016-7037(95)00011-9.

[ref55] Schmidt M. P.; Ilott A. J.; Phillips B. L.; Reeder R. J. Structural Changes upon Dehydration of Amorphous Calcium Carbonate. Cryst. Growth Des. 2014, 14, 938–951. 10.1021/cg401073n.

[ref56] Goldsmith J. R.; Graf D. L. Relation between Lattice Constants and Composition of the Ca-Mg Carbonates. Am. Mineral. 1958, 43, 84–101.

[ref57] Lumsden D. N. Discrepancy between Thin-Section and X-Ray Estimates of Dolomite in Limestone. J. Sediment. Res. 1979, 49, 429–435. 10.1306/212F7761-2B24-11D7-8648000102C1865D.

[ref58] Kaczmarek S. E.; Sibley D. F. On the Evolution of Dolomite Stoichiometry and Cation Order during High-Temperature Synthesis Experiments: An Alternative Model for the Geochemical Evolution of Natural Dolomites. Sediment. Geol. 2011, 240, 30–40. 10.1016/j.sedgeo.2011.07.003.

[ref59] Goldsmith J. R.; Graf D. L. Structural and Compositional Variations in Some Natural Dolomites. J. Geol. 1958, 66, 678–693. 10.1086/626547.

