# Supplementary material for: Heat-Induced Dolomitization of Amorphous Calcium Magnesium Carbonate in a CO2-Filled Closed System
Source: ACS Omega. 2022 Nov 28;7(49):44670–6. doi: 10.1021/acsomega.2c03258 (PMC9753508; doi:10.1021/acsomega.2c03258)
Supplement: Supplementary file 1 — ao2c03258_si_001.pdf [file ao2c03258_si_001.pdf]

**Supporting Information for**

# Heat-Induced Dolomitization of Amorphous Calcium Magnesium Carbonate (ACMC) in a $CO_2$-Filled Closed System

Shingo Sugawara[a]*, Wataru Fujiya[a], Hiroyuki Kagi[b], Akira Yamaguchi[c], and Ko Hashizume[a]

[a]Faculty of Science, Ibaraki University, 2-1-1 Bunkyo, Mito, Ibaraki 310-8512, Japan

[b]Geochemical Research Center, Graduate School of Science, The University of Tokyo, 7-3-1 Hongo, Tokyo 113-0033, Japan

[c]National Institute of Polar Research, 10-3 Midori-cho, Tachikawa, Tokyo 190-8518, Japan

*Email: 21nd202t@vc.ibaraki.ac.jp

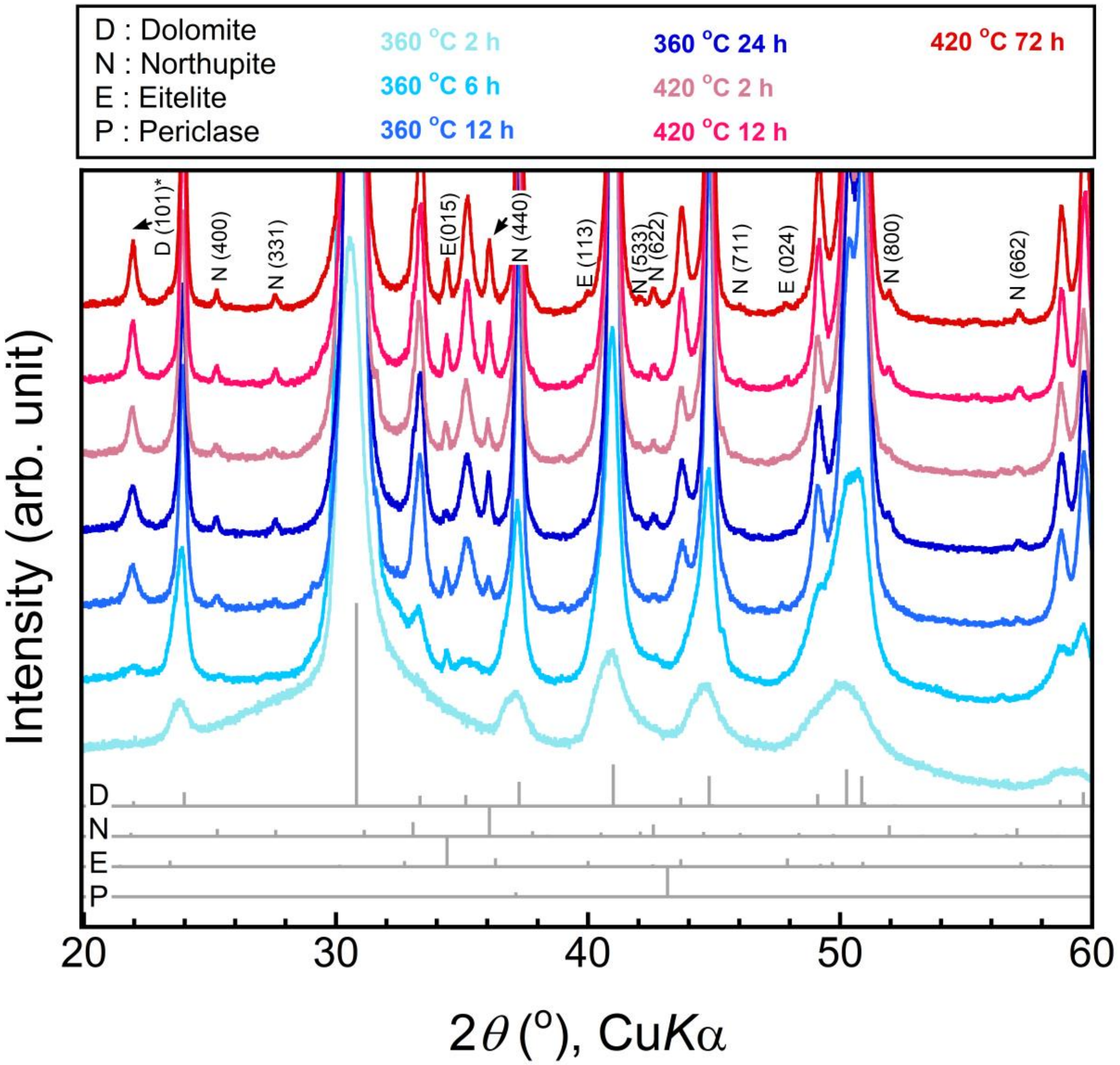


**Figure S1.** Enlarged views of Figure 4 showing the XRD patterns of the samples heated in a closed system filled with $CO_2$ gas. The phase identification and indexing of the XRD patterns were carried out using COD files 9004933 (dolomite), 9009581 (northupite [$Na_3Mg(CO_3)_2Cl$]), 9000298 (eitelite [$Na_2Mg(CO_3)_2$]), and 9006786 (periclase [MgO]). The cation ordering reflections are indicated by an asterisk.

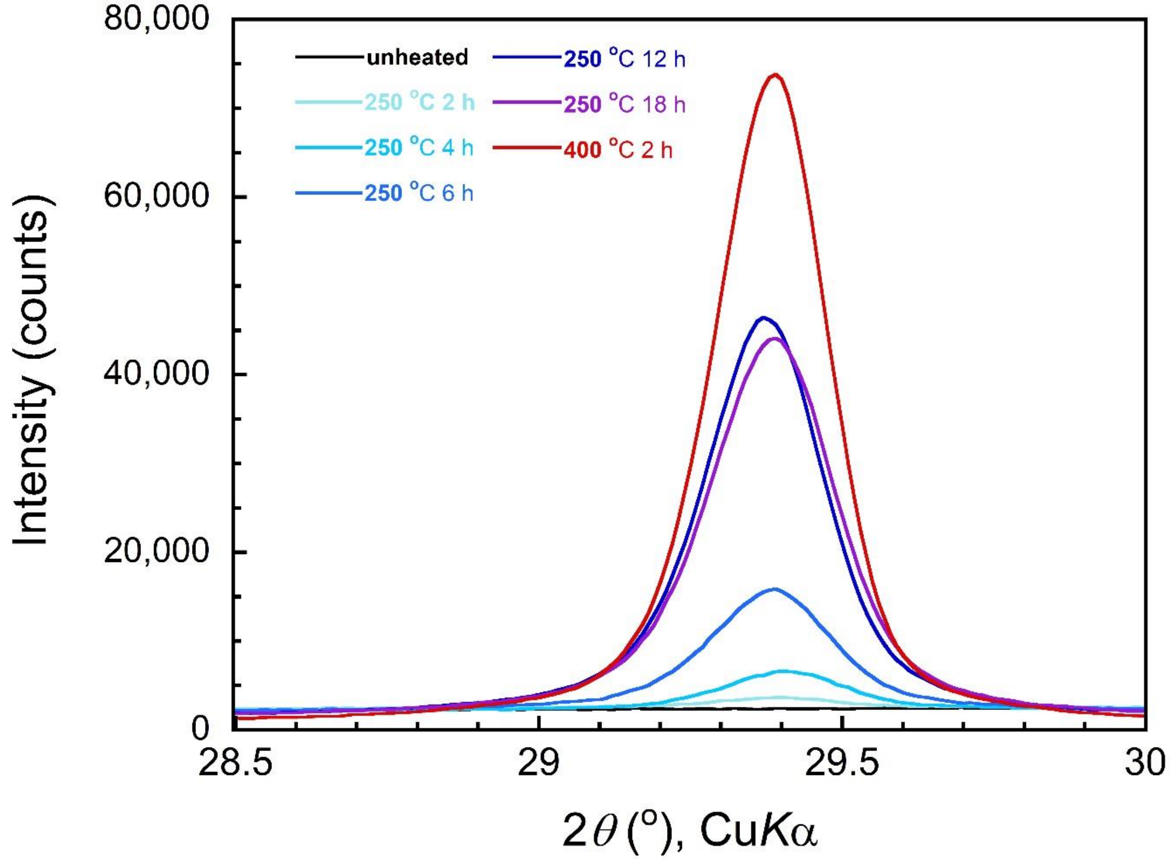


**Figure S2.** XRD patterns of the ACC (unheated) and the samples heated in air. There is no systematic variation in peak positions among the samples.

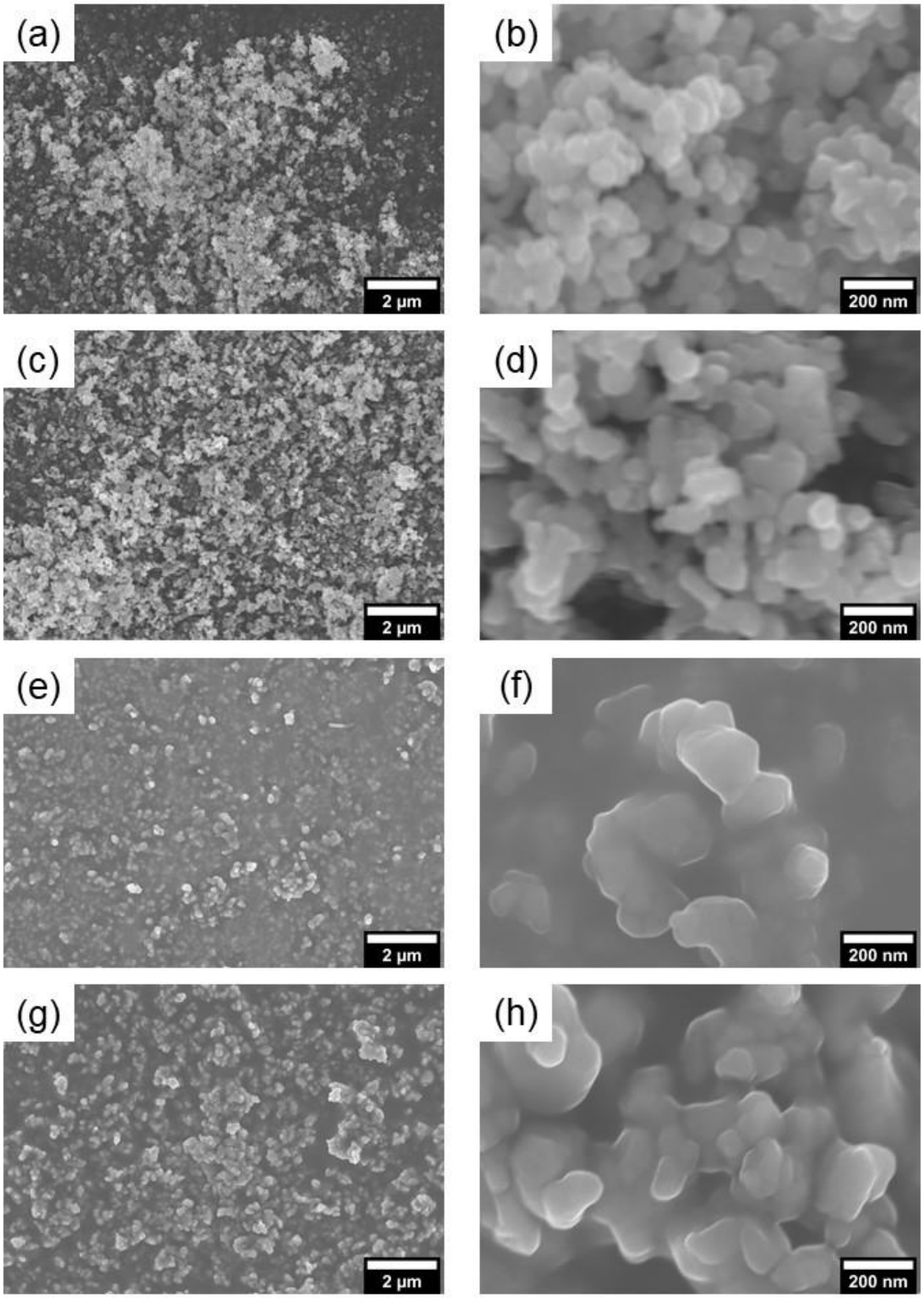
(a)
2 μm
(b)
200 nm
(c)
2 μm
(d)
200 nm
(e)
2 μm
(f)
200 nm
(g)
2 μm
(h)
200 nm

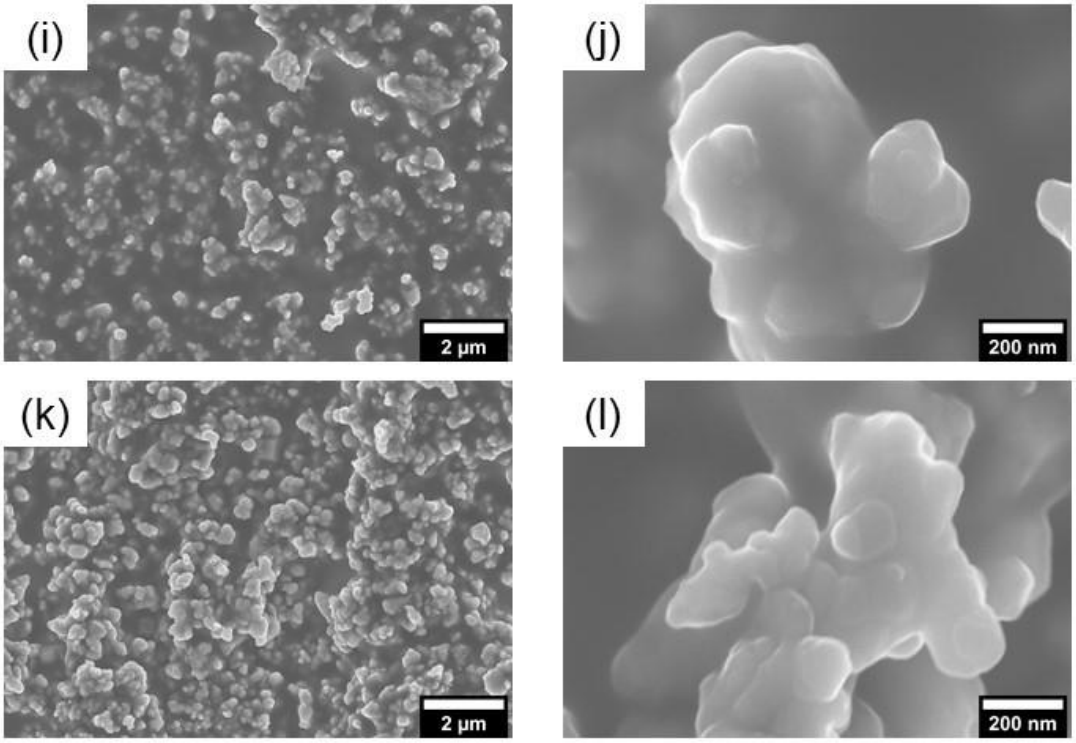


**Figure S3.** Secondary electron images of the samples heated at 360 °C for (a) 2 h, (c) 6 h, (e) 12 h, and (g) 24 h, and at 420 °C for (i) 2 h and (k) 12h in a closed system filled with $CO_2$ gas. (b), (d), (f), (h), (j), and (l): Enlarged views of (a), (c), (e), (g), (i), and (k), respectively.

**Thermogravimetry and differential thermal analysis (TG-DTA)**

To investigate dehydration, crystallization, and decomposition temperatures, we conducted a TG-DTA measurement (Rigaku TG-DTA8122 at Ibaraki University) using 5 mg of the synthesized ACMC (Figure 1). The measurement was performed from room temperature to 1000 °C at a heating rate of 10 °C $min^{-1}$ under an $N_2$ flow (130 mL $min^{-1}$). The reference was 5 mg of alumina powder.

**Powder X-ray diffraction (XRD) measurements**

We performed powder XRD analyses using an X-ray diffractometer (Rigaku Miniflex 600 at Ibaraki University) and a pure silicon plate with a low background. The measurement condition of XRD was 0.01° step and a scanned region from 10° to 70° in 2θ with CuKα ($K\alpha_1$, λ = 1.54059 Å) radiation operated at 15 mA and 40 kV. A nickel (Ni) Kβ filter was used to monochromatize X-rays. The peak positions were calibrated using a silicon standard (NIST SRM 640f). The peak positions of 104 reflection ($K\alpha_1$) in all samples was obtained by curve fitting with a Gaussian function (Table 1).

**Electron probe microanalysis**

We measured the Mg/Ca ratio of the sample, which was washed by ultrapure water after heating at 420 °C for 72 h, using an electron probe microanalyzer (JEOL JXA-8200 at NIPR) with a 15 kV/5 nA electron beam of 10 μm in diameter. The standards were calcite [$CaCO_3$] (Ca) and olivine [$(Mg, Fe)_2SiO_4$] (Mg).

The sample was put in a tungsten carbide piston cylinder of 4 mm in inner diameter and then pressed using a hydraulic press to produce a pellet. The pellet was embedded in resin, and then, its surface was polished. The polished sample was coated with carbon.

**Field-emission scanning electron microscope observations**

We observed the dehydrated ACMC and heated samples using a field-emission scanning electron microscope (Hitachi High-Tech S-4800 at Ibaraki University). The dehydrated ACMC and heated samples were put on carbon tapes and fixed on a glass slide. and then coated with carbon prior to the observation.

**The crystallization process of amorphous calcium carbonate (ACC) by heating in air**

ACC was synthesized by mixing ice-cooled 10 mL 0.1 M $CaCl_2$ solution and 10 mL 0.1 M $Na_2CO_3$ solution.[1,2] After mixing, the precipitate was dried in the same manner as that for ACMC. Then, the ACC samples were crystallized to calcite by heating at 250 °C for 2, 4, 6, 12, and 18 h, and at 400 °C for 2 h.

Figure S2 shows XRD patterns of the samples. The peak positions of the samples showed no systematic variation with increasing heating time. These results may indicate that the systematic peak shift observed for the heated ACMC was not attributed to the variable heating time, but due to the changes in the Mg/Ca ratio.

**References**


(1) Koga, N.; Nakagoe, Y.; Tanaka, H. Crystallization of Amorphous Calcium Carbonate. Thermochim. Acta 1998, 318, 239–244.

(2) Yoshino, T.; Maruyama, K.; Kagi, H.; Nara, M.; Kim, J. C. Pressure-Induced Crystallization from Amorphous Calcium Carbonate. Cryst. Growth Des. 2012, 12, 3357–3361.
